# Tumor-targeted SN38 inhibits growth of early stage non-small cell lung cancer (NSCLC) in a KRas/p53 transgenic mouse model

**DOI:** 10.1371/journal.pone.0176747

**Published:** 2017-04-28

**Authors:** Alexander Y. Deneka, Leora Haber, Meghan C. Kopp, Anna V. Gaponova, Anna S. Nikonova, Erica A. Golemis

**Affiliations:** 1 Program in Molecular Therapeutics, Fox Chase Cancer Center, Philadelphia, Pennsylvania, United States of America; 2 Department of Biochemistry, Kazan Federal University, Kazan, Russia; 3 Program in Molecular and Cell Biology and Genetics, Drexel University College of Medicine, Philadelphia, Pennsylvania, United States of America; 4 Laboratory of Genome Engineering, Moscow Institute of Physics and Technology, Dolgoprudny, Russia; 5 Immanuel Kant Baltic Federal University, Konigsberg, Russia; Emory University Winship Cancer Institute, UNITED STATES

## Abstract

Non-small cell lung cancer (NSCLC) is the leading cause of cancer death worldwide, with a 5-year survival of only ~16%. Potential strategies to address NSCLC mortality include improvements in early detection and prevention, and development of new therapies suitable for use in patients with early and late stage diagnoses. Controlling the growth of early stage tumors could yield significant clinical benefits for patients with comorbidities that make them poor candidates for surgery: however, many drugs that limit cancer growth are not useful in the setting of long-term use or in comorbid patients, because of associated toxicities. In this study, we explored the use of a recently described small molecule agent, STA-8666, as a potential agent for controlling early stage tumor growth. STA-8666 uses a cleavable linker to merge a tumor-targeting moiety that binds heat shock protein 90 (HSP90) with the cytotoxic chemical SN38, and has been shown to have high efficacy and low toxicity, associated with efficient tumor targeting, in preclinical studies using patient-derived and other xenograft models for pancreatic, bladder, and small cell lung cancer. Using a genetically engineered model of NSCLC arising from induced mutation of KRas and knockout of Trp53, we continuously dosed mice with STA-8666 from immediately after tumor induction for 15 weeks. STA-8666 significantly slowed the rate of tumor growth, and was well tolerated over this extended dosing period. STA-8666 induced DNA damage and apoptosis, and reduced proliferation and phosphorylation of the proliferation-associated protein ERK1/2, selectively in tumor tissue. In contrast, STA-8666 did not affect tumor features, such as degree of vimentin staining, associated with epithelial-mesenchymal transition (EMT), or downregulate tumor expression of HSP90. These data suggest STA-8666 and other similar targeted compounds may be useful additions to control the growth of early stage NSCLC in patient populations.

## Introduction

Non-small cell lung cancer (NSCLC) is the leading cause of cancer-related death in the world, and metastasis is the most common cause of death in lung cancer patients [1]. In the United States, approximately 160,000 patients died of NSCLC in 2013 [2]. NSCLC typically has a poor prognosis if tumors have begun to disseminate from the original site, with 5-year survival rate of 17.4% for patients diagnosed with stage 3 tumors, and 4% if diagnosed with stage 4 tumors [1]. These advanced NSCLC tumors typically respond initially but subsequently develop resistance to chemotherapies and targeted therapies. In contrast, NSCLC tumors can often be treated successfully through surgery and radiotherapy if detected at an early stage, with the 5-year survival rates 45% and 30% for stages 1 and 2, respectively [3]. However, for many patients, potential curative treatment of early tumors is challenging or impossible because co-morbidities or other issues preclude the use of surgery or irradiation [1]. In these cases, the availability of well-tolerated reagents suitable for slowing tumor growth would be expected to yield significant health benefits.

For both early and advanced NSCLC (and other tumors), a critical barrier to effective treatment of tumors with chemotherapies has been the inability to concentrate cancer drugs in the tumor at sufficient levels to achieve therapeutic benefit without simultaneously inducing untenable degrees of toxicity in normal tissues. Towards this end, extensive effort has been devoted to developing targeting strategies to concentrate cytotoxic agents within tumors. Two recent studies described STA-8666 as a new class of tumor-targeted agent [4, 5]. STA-8666 is a tripartite molecule in which a moiety targeting activated Heat Shock Protein 90 (HSP90) is fused via a cleavable chemical linker to the cytotoxic compound SN38, the active metabolite of irinotecan, which inhibits topoisomerase I to induce double strand and single strand DNA breaks [6]. Because of its critical role as a chaperone stabilizing and enhancing the activity of multiple client proteins that exhibit fragility in cells growing under stressful conditions, HSP90 is highly upregulated and activated in tumor tissue [7, 8], and typically cannot be downregulated without significantly impairing tumor growth. Pharmacokinetic profiling of STA-8666 demonstrated high ratios of STA-8666 and the cleaved SN38 metabolite in tumor versus normal tissue or plasma (ranging from 7.5:1 to 20:1, [4, 5]), and longer half-life of SN38 in tumors [5]. These facts suggested it might be useful as an agent to control the growth of early stage NSCLC.

The most common causes of NSCLC in humans are somatic activating mutations in *KRAS*, which occur in approximately 30% of human lung adenocarcinomas [9] and loss of the tumor suppressor *TP53* (60% of all cases) [10]. In the transgenic *KRas*^*tm4Tuj*^*/Trp*^*53tm1Brn*^ mouse model [11, 12], inhalation of adenovirus bearing the Cre recombinase [13] induces activating mutation of *KRas* in parallel with inactivation of *Trp53*. In this system, introduction of adenovirally-borne *Cre* at post-natal week 10 results in development of lung adenomas at ~ 5–5.5 months of age (~22 weeks), progressing to adenocarcinomas and leading to severe lung tumor burden at 6–7 months of age [11], with tumors that conserve many pathological and molecular features of human NSCLC [14]. Using this model, we have investigated whether continuous administration of STA-8666 from immediately after the induction of tumors for 4 months is well-tolerated and effective in controlling the growth of early stage NSCLC, and explored the mechanism of action of STA-8666.

## Materials and methods

### Mouse strains and drug treatment

The Institutional Animal Care and Use Committee (IACUC) of Fox Chase Cancer Center prospectively approved all experiments involving mice (animal protocol #14–06). *In vivo* experiments used a conditional *KRas*^*tm4Tuj*^*/Trp*^*53tm1Brn*^ mice model with Adeno-cre activated lung tumor growth, following published protocols [11, 12]. For infection, mice were anesthetized with ketamine (100ng/ml)/xylazine (100mg/ml) in a 10:1 mixture diluted 10-fold in 0.9% NaCl and injected intraperitoneally (to 1% of body weight). Once mice were anesthetized, an Excel Safelet Catheter (24G x ¾”) (EXELINT, Los Angeles, CA) was inserted through the oral cavity into the trachea. Adenoviral activation of oncogenic *KRas* and inactivation *Trp53* was induced by introduction of adenovirus (2.5^e6^ viral particles in 75μl diluted in MEM media and CaCl_2_) into the trachea. Diluted virus was disseminated through the lungs as a result of normal inhalation. After infection, mice were placed into clean cages and handled as biohazards for two weeks. Treatment with STA-8666 (Synta Pharmaceuticals Corp., Lexington, MA) or vehicle commenced 1 week after adenovirus administration. STA-8666 was formulated in 10% DMSO/Cremaphor saline. For vehicle, we used 10% Cremaphor in 5% dextrose. Weekly retroorbital (r.o.) dosing with 150 mg/kg STA-8666 or vehicle was performed for 15 weeks, from 11–26 weeks of age. At 27 weeks of age, all mice in the study were euthanized by CO_2_ inhalation and tissues used for further analysis.

### Magnetic-resonance imaging (MRI) and image analysis

MRI of murine lungs and tumors was performed for vehicle versus STA-8666 treated mice at 22 and 26 weeks of age. For MRI, mice were anesthetized with 1–2% isoflurane in O_2_, and imaged using a vertical bore 7 Tesla magnet, Bruker DRX300 spectrometer, ParaVision 3.0.2 software (Bruker), and a single tuned ^1^H cylindrical radiofrequency coil, using protocols described in detail in [15]. Tumor volumes were quantified using Image J software [16]. For estimation of lung tumor volume, the tumor images were manually selected, to exclude adjacent heart and blood vessels. The products of area measurements, number of contiguous images and slice thickness were summed as in [17].

### Tissue preparation and histology

Lungs were collected at a treatment endpoint predefined as 27 weeks of age. For tissue preparation, lungs were fixed in 10% phosphate-buffered formaldehyde for 24–48 hrs, dehydrated by incubation in ethanol followed by xylene (70% ethanol, 3 hr; 95% ethanol, 2 hr; 100% ethanol, 2 hr; ethanol-xylene, 1hr; xylene, 3hr) then embedded in paraffin. Paraffin blocks were cut into 5 μm sections, mounted on microscope slides, and stored at room temperature until used. Prepared specimens were stained with hematoxylin and eosin (H&E) (Sigma-Aldrich, St. Louis, MO) and analyzed by immunohistochemistry (IHC) or immunofluorescence using standard protocols. Antibodies used in IHC were to Ki-67 (DAKO, Carpinteria, CA), cleaved caspase 3 (#9664L, Cell Signaling, Beverly, MA), pS^824^-KAP1 (ab70369, Abcam, Cambridge, MA), pT^202^/Y^204^-ERK1/2 (#4996S Cell Signaling, Beverly, MA) and HSP90 (NB120-2928, Novus Biologicals, Littleton, CO). For immunofluorescence, we used antibody to vimentin (ab92547, Abcam, Cambridge, MA) as primary reagent and secondary Alexafluor 568 tagged donkey anti-rabbit antibody (Life Technologies, Eugene, OR), and counterstained with a 2 μmol/L 4′, 6-diamidino-2-phenylindole (DAPI) (#1652731, Life Technologies, Eugene, OR) solution to visualize DNA.

### Quantification of IHC and immunofluorescence

Stained slides were scanned with Vectra Automated Quantitative Pathology Imaging System (Perkin Elmer, Waltham, MA). The number of independent tumors and average size of individual tumors in lungs from mice treated with STA-8666 or vehicle were obtained using Image J software [16]. Expression levels of Ki-67, caspase 3, pS^824^-KAP1, pT^202^/Y^204^-ERK1/2, HSP90 and vimentin were quantified using Vectra protocols and algorithms specified by the manufacturer. H-scores were calculated as follows: the percentage of cells at each staining intensity level was calculated, and an H-score was assigned and calculated for each slide using the following formula: [1 × (% cells 1+) + 2 × (% cells 2+) + 3 × (% cells 3+)] as described [18, 19].

### Cell lines and proliferation assay

The A549 and H441 human NSCLC cell lines were obtained from the American Type Culture Collection (ATCC). Cells (1 × 103 cells/well) were plated in RPMI1640 (H441) or DMEM (A549) media with 10% FBS in 96-well cell culture plates. Vehicle (0.1% DMSO), STA-8666 or irinotecan were added in concentrations of 0.1, 1 and 10 μM to each well, and cells were grown for up to 5 days, in duplicate for each of 5 assay time points. On days 1, 2, 3, 4, or 5 after plating, *CellTiter*-*Blue*^®^ (Promega, Fitchburg, WI) reagent was added to all wells; after 4 hours incubation at 37°C, optical density readings were made in the 570–600 and 420–480 nm wave-length ranges, respectively, using *Perkin*-Elmer ProXpress Visible-UV-fluorescence 16 bit scanner (Perkin-Elmer, Waltham, MA). Values from at least two independent experiments were averaged.

### Western blot analysis

A549 and H441 cells were plated in RPMI1640 (H441) and DMEM (A549) media with 10% FBS in 6-well cell culture plates. After cells attached, vehicle (0.1% DMSO), STA-8666 and irinotecan were added at 0.1, 1 and 10 μM. Adherent cells were lysed in CelLytic MT Cell Lysis Reagent (Sigma-Aldrich, St. Louis, MO) at 24, 48 and 72 hr time points. Protein concentrations of the resulting lysates were measured using the Pierce BCA Protein Assay Kit (Thermo Scientific, Waltham, MA). Western Blotting was performed using standard procedures, and blots developed by chemiluminescence using Luminata Western HRP substrates (Classico, Crescendo EMD Millipore) and Immun-Star AP Substrate (Bio-Rad Laboratories).

Primary antibodies were used at a 1:1000 dilution and included: anti-PARP (#9542L Cell Signaling, Beverly, MA), anti-pT^202^/Y^204^-ERK1/2 (#4996S Cell Signaling, Beverly, MA), anti-Erk1/2, (#4696S Cell Signaling, Beverly, MA), anti-p^T308^Akt, (#2965S Cell Signaling, Beverly, MA), anti-Akt, (#2920S Cell Signaling, Beverly, MA), anti-p^T68^Chk2, (#MAB1626 R&D Systems, Minneapolis, MN); anti-Chk2, (#3440 Cell Signaling, Beverly, MA), anti-p^Y15^CDK1, (#9111L Cell Signaling, Beverly, MA), anti-CDK1, (#SC54 Santa Cruz Biotechnology, Dallas, TX); anti-E-cadherin, (#3195S Cell Signaling, Beverly, MA); anti-vimentin, (ab92547, Abcam, Cambridge, MA), anti-Snail, (ab180714, Abcam, Cambridge, MA), and anti-vinculin (mouse, monoclonal hVIN-1, #V9131, Sigma-Aldrich, St. Louis, MO). Secondary anti-mouse and anti-rabbit HRP-conjugated antibodies (GE Healthcare, Little Chalfont, UK) were used at a dilution of 1:10,000, and secondary anti-mouse and anti-rabbit AP-conjugated antibodies (Jackson Immunoresearch Labs, West Grove, PA) were used at a dilution of 1:5,000. Quantification of signals on Western blots was done using the NIH ImageJ Imaging and Processing Analysis Software [16].

### Statistical and correlation analysis

We used a nonparametric Mann-Whitney t-test to calculate p-values to compare differences in markers’ expression between treatment cohorts. For correlation analysis we used a Spearman nonparametric correlation coefficient with two-tailed p-value. All statistical analysis was performed in GraphPad Prism software.

## Results

### STA-8666 reduces the number and size of tumors forming in the KRas^tm4Tuj^/Trp^53tm1Brn^ mouse model

**Fig 1:**
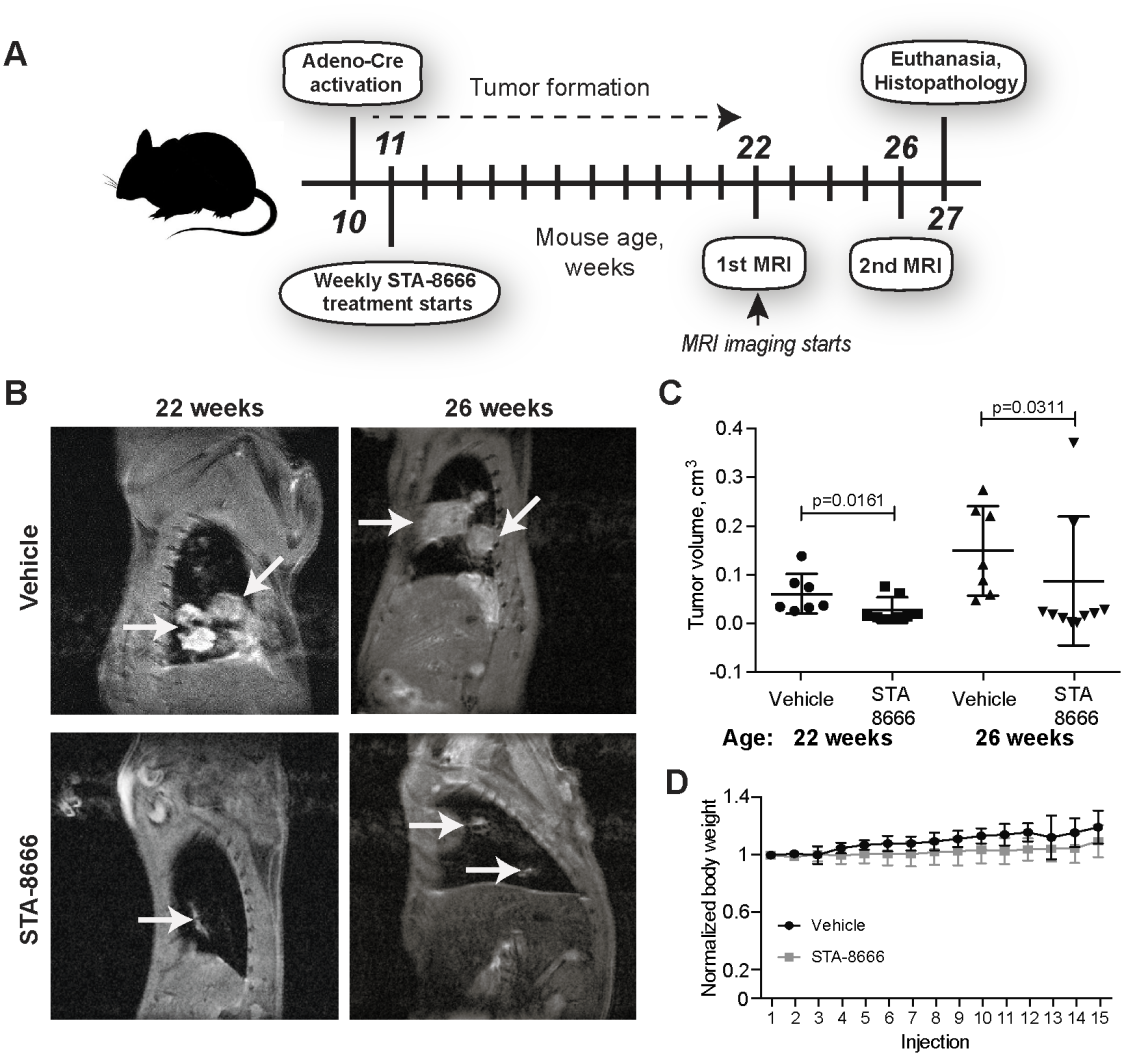
Adeno-cre induced murine lung cancer model. **A**. Experimental design. **B**. Representative MRI images of murine lungs and tumors (arrows) from the vehicle and STA-8666 treatment cohorts. **C**. Quantified tumor volume determined by MRI in vehicle versus STA-8666 treated mice. **D**. Body weight dynamics in mice receiving vehicle or STA-8666.

The outline of *in vivo* experiments is shown in Fig 1A. We induced formation of NSCLC in the transgenic *KRas*^*tm4Tuj*^*/Trp*^*53tm1Brn*^ mouse model [11, 12] by inhalation of adenovirus bearing the Cre recombinase at post-natal week 10 [13], causing activating mutation of *KRas* in parallel with inactivation of *Trp53*. Beginning at 11 weeks of age, mice received weekly injections of 150 mg/kg STA-8666 or of vehicle. Using magnetic resonance imaging (MRI) (Fig 1B), we assessed tumor growth at weeks 22 and 26 of age. STA-8666 caused a statistically significant decrease in tumor growth at both time points (Fig 1C). At 26 weeks, notably, the average tumor volume in vehicle treated mice was 0.16 cm^3^, while in STA-8666 mice it was 0.098 cm^3^ (p = 0.03). Further, comparison of individual mice indicated that almost all of the STA-8666 mice had little if any discernible increase in tumor volume between 22 and 26 weeks, while tumor volumes in many of the vehicle-treated mice had increased in this interval. Parallel weekly analysis of body weight indicated no differences between mice dosed with vehicle and STA-8666, while visual inspection indicated no signs of distress (Fig 1D).

**Fig 2:**
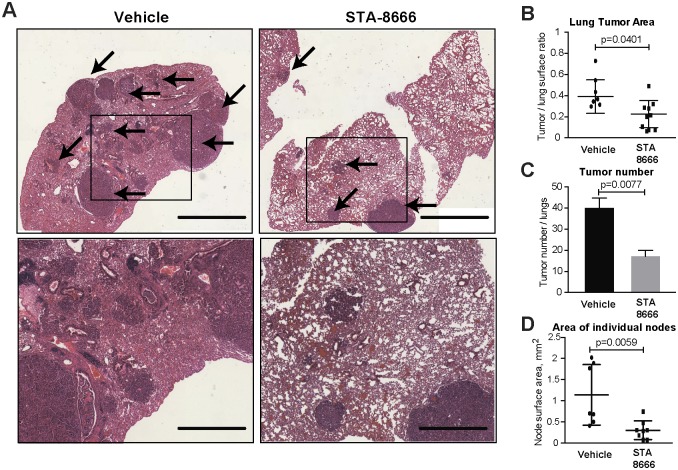
STA-8666 treatment prevents tumor growth *in vivo*. **A**. Representative H&E stained lungs. Arrows indicate tumors. Magnification: 4x. Scale bars: 2mm, top panels; 850 μm, bottom panels. **B.-D**. Quantification of total tumor surface area (**B**), number of independent tumors (**C**), and average size of individual tumors (**D**).

The tumors developing in this mouse model typically progress to adenocarcinomas and mice experience a severe lung tumor burden at 26–30 weeks of age [11]. Therefore, at 27 weeks of age, all mice in the study were euthanized, and tissues used for histopathological analysis. H&E staining of lungs indicated greater number and size of tumors in vehicle-treated versus STA-8666-treated mice (Fig 2A). A Vectra automated quantitative pathology imaging system was used to quantify the total area, numbers, and size of tumors for each lung. STA-8666 treatment significantly reduced overall tumor surface area, with a ratio of tumor to normal lung tissue of ~2:5 in vehicle-treated mice versus ~1:5 in STA-8666-treated mice (p = 0.04) (Fig 2B). STA-8666 strikingly reduced the number of independent tumor foci detectable in the lungs, with quantified values of 40±5 in vehicle-treated mice versus 18±3 in STA-8666-treated mice (p = 0.0077) (Fig 2C), as well as the average area of individual tumor nodes from 1.2 mm^2^ in vehicle-treated mice to 0.3 mm^2^ in STA-8666-treated mice (p = 0.0059) (Fig 2D). We also note, for the analysis of signaling described below, values obtained are likely to be an underestimate of STA-8666 activity, as for 3 (of 10) mice treated with STA-8666, no tumors were detectable in the lungs upon euthanasia, making it impossible to perform IHC in these apparent strong responders.

### STA-8666 reduces cell division, and induces apoptosis in tumors

**Fig 3:**
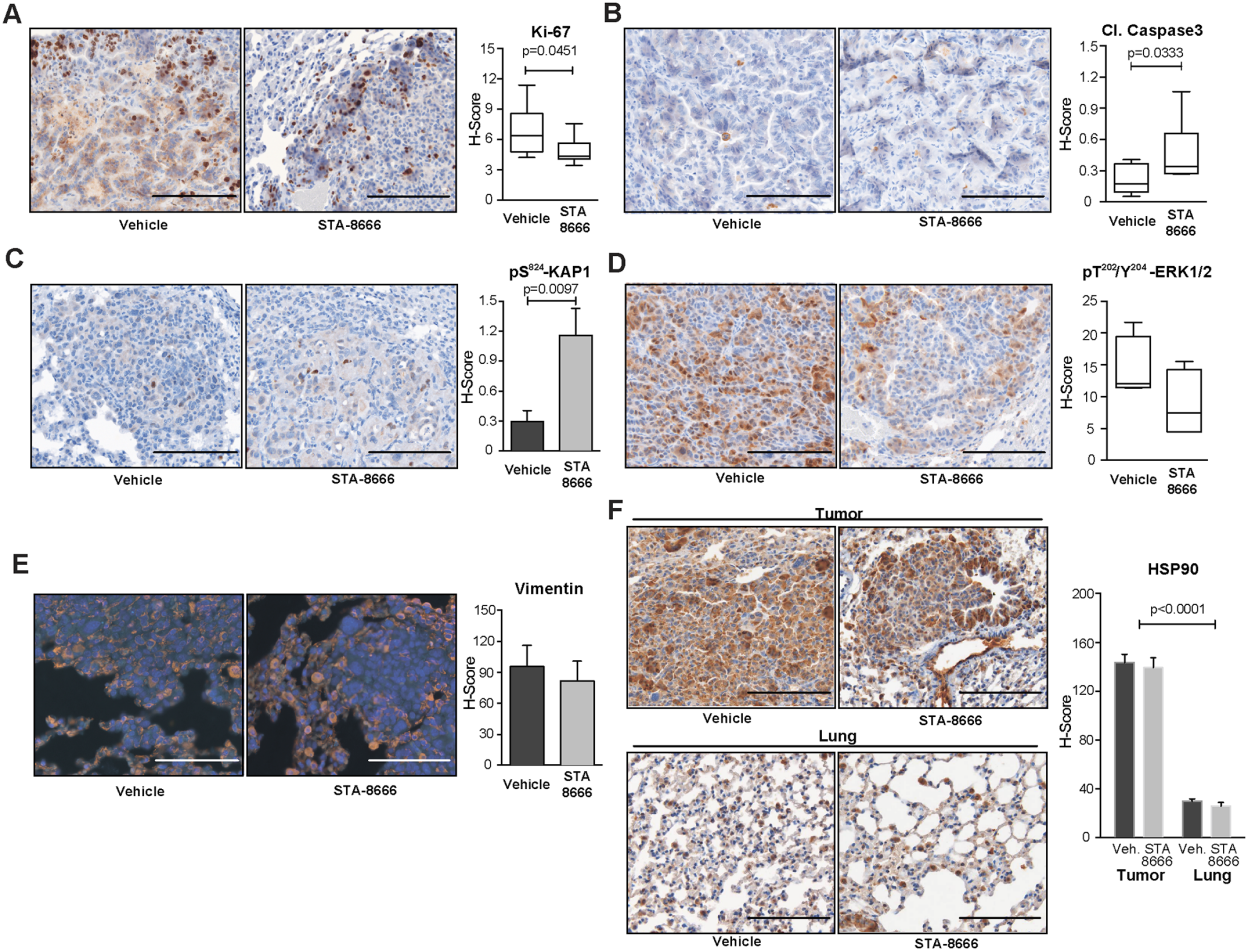
Signaling consequences of STA-8666 treatment. **A.-F**. Left, Representative images and right, quantification of IHC or immunofluorescence for Ki-67 (**A**), cleaved caspase 3 (**B**), pS^824^-KAP1 (**C**), pT^202^/Y^204^-ERK1/2 (**D**), vimentin (light red) and DAPI (blue) (**E**) and HSP90 (**F**). Magnification: 20x. Scale bars: 75 μm, except for vimentin, where scale bar is 40 μm.

For a more detailed phenotypic description of STA-8666 activity, we performed IHC analysis of lungs from the experimental cohorts, separately quantifying results for tumor and normal tissue. Staining with the cell division marker Ki-67 indicated the proliferative index of tumors (presented as H-score, [18, 19]) was significantly reduced (p = 0.045) by STA-8666 relative to vehicle treatment (Fig 3A). By contrast, in non-tumor tissue, fewer than 0.01% of cells were positive for Ki-67 staining, and no differences related to drug treatment were observed (panel A in S1 Fig). Caspase-3 cleavage is an indicator of apoptosis, the most common form of cell death in tumors [20]. STA-8666 also significantly increased caspase-3 cleavage in tumors compared to vehicle, with an H-score of 0.2 versus 0.4 for vehicle-treated lungs (p = 0.033) (Fig 3B). Almost no cleaved caspase 3 was detected in normal lung tissue (panel B in S1 Fig).

### STA-8666 induces DNA damage response signaling and inhibits proliferative signaling in tumors

We explored the mechanism of action of STA-8666 in tumors. The primary mechanism of action reported for SN38, the active metabolite of STA-8666, is inhibition of topoisomerase I, which triggers production of single- and double-stranded DNA breaks [21]. KAP1 is a mediator of DNA damage signaling following induction of DNA lesions [22], and SN38 treatment elevates the level of KAP1 activation (indicated by phosphorylation on serine 824, pS^824^-KAP1) [22]. We analyzed pS^824^-KAP1 in tumors and normal lung tissue treated with STA-8666 or vehicle (Fig 3C). Quantitative Vectra analysis revealed a dramatic increase in expression of this marker in tumor tissue treated with STA-8666 (a 3.9-fold increase versus vehicle: p = 0.0097). Importantly, overall levels of pS^824^-KAP1 staining were very low in untransformed lung tissue (with detectable signal in fewer than 0.0025% of lung cells) and did not differ between STA-8666 and vehicle-treated lung tissue (panel C in S1 Fig), implying drug action was largely restricted to tumor tissue.

We also investigated how treatment with STA-8666 interfered with essential tumor-related signaling. KRas/p53 lung adenocarcinomas have significant elevation of signaling through Extracellular signal-Regulated Kinases (ERKs) [23]. IHC analysis of lung tissue stained with the antibodies against activated pT^202^/Y^204^-ERK1/2 suggests decreased activation of this signaling pathway within tumors in the STA-8666-treated cohort, compared to vehicle (H scores of 12 versus 7.6), although these differences did not achieve statistical significance (p = 0.177) (Fig 3D). In contrast, levels of positive pT^202^/Y^204^-ERK1/2 staining in non-affected lung tissue were below detection limits (panel D in S1 Fig).

As an extended hypothesis for STA-8666 action, we considered the possibility that this compound might affect the process of epithelial-mesenchymal transition (EMT), as tumors with a greater proportion of epithelial cells are more sensitive to treatment with chemotherapeutic agents [24, 25]. Vimentin is a marker of mesenchymal identity, decreased in tumors that have become more epithelial [26]. Immunofluorescence-based detection of vimentin followed by Vectra analysis indicated no significant changes in numbers of cells positive for this protein, or degree of vimentin expression in individual cells, between STA-8666 and vehicle-treated cohorts (Fig 3E). These data indicated that differences in tumor volume induced by STA-8666 were unlikely to be related to the epithelial versus mesenchymal identity of the tumor cells.

We also analyzed HSP90 expression in tumor versus normal lung tissue (Fig 3F). Reflecting the difficulty in downregulating HSP90 in tumors, expression of this protein was essentially equivalent in tumors of mice treated with STA-8666 or vehicle. HSP90 expression was highly elevated in tumor tissue versus normal lung tissue of both treatment groups (p<0.0001 for each cohort) (Fig 3F). Coupled with the fact that tumor-associated HSP90 is in an activated configuration that is more effectively bound by STA-8666 (see discussion in [5]), this observation supported the interpretation that the observed difference in KAP1 and ERK activation reflected concentration of STA-8666 in tumors.

### Correlation of response indicators

**Fig 4:**
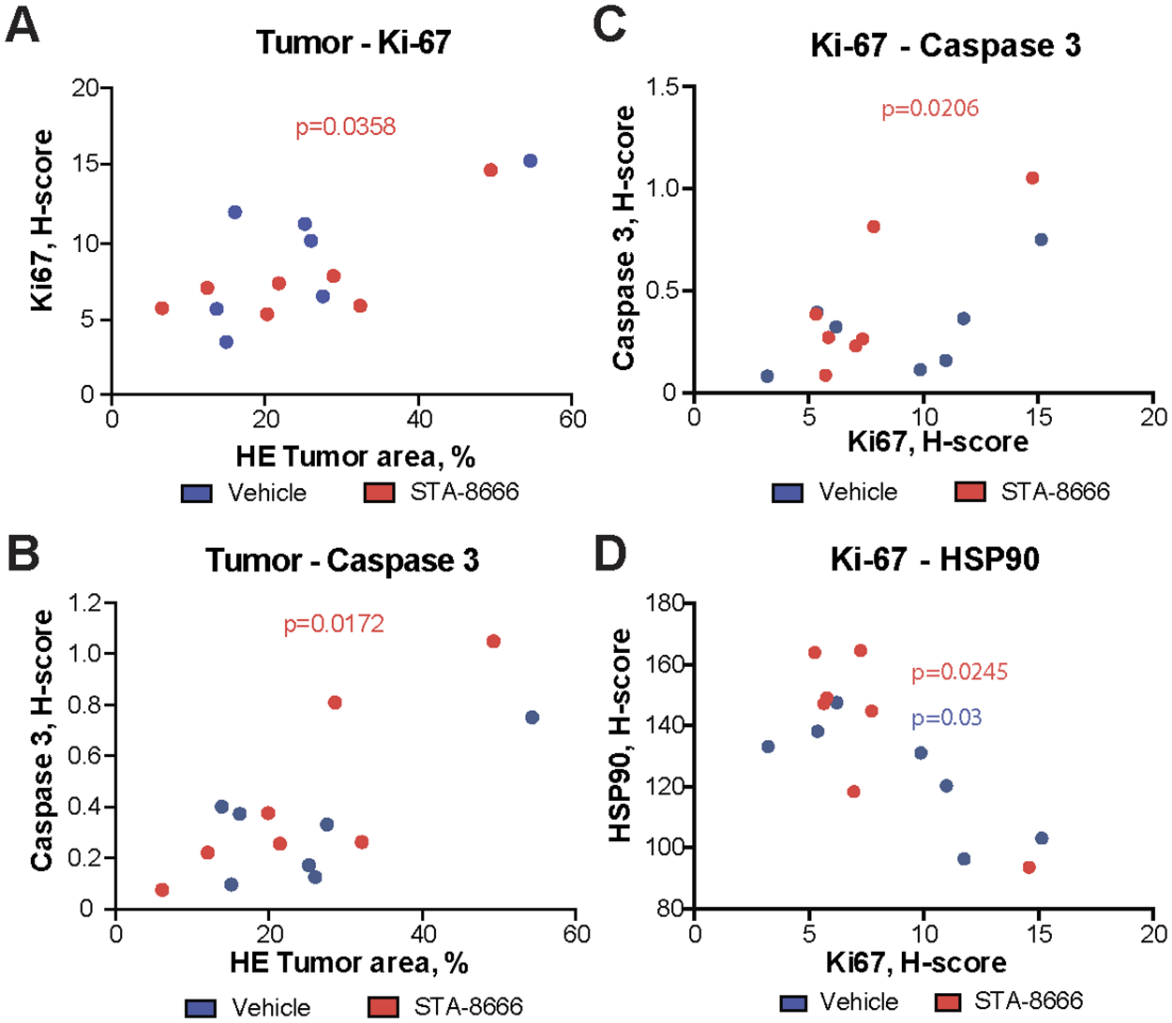
Statistical analysis of correlations in between markers expression in vehicle and STA-8666 treated mice cohorts. **A-D**. Shown Spearman nonparametric correlation coefficient with two-tailed p-value between H-score for Ki-67 and tumor area (**A**), H-score for cleaved caspase 3 and tumor area (**B**), H-scores for Ki-67 and cleaved caspase 3 (**C**), and H-scores for Ki-67 and HSP90 (**D**). Each dot represents an individual mouse. P values shown in red are for STA-8666 treatment cohort, for blue are for vehicle treatment cohort.

Heterogeneity of response is common when performing *in vivo* experiments on murine models, complicating assessment of which biomarkers are most likely functionally correlated with outcomes. We performed correlative statistical analysis for all of the parameters described above (Fig 4, S2 Fig). We found that larger tumor volumes directly correlated with expression of the proliferation marker Ki-67 and the apoptotic marker cleaved caspase 3 in the STA-8666 treated cohort (p = 0.0358 and p = 0.0172, respectively) (Fig 4A and 4B). As expected from this somewhat surprising outcome, Ki-67 and cleaved caspase 3 levels also correlated within individual STA-8666-treated mice (Fig 4C). In contrast, no significant correlation was observed for Ki-67, cleaved caspase 3, or tumor size in vehicle-treated animals (Fig 4A, 4B and 4C). Together, these results suggested that reduction in proliferation, rather than increase in apoptotic frequency, was more relevant to control of tumor growth. Finally, in comparative analysis of all of the signaling parameters we had assessed, we found that Ki-67 staining negatively correlated with expression of HSP90 (p = 0.0245 and p = 0.03 for the STA-8666- and vehicle-treated groups respectively) (Fig 4D). This was compatible with the interpretation that tumors having higher levels of HSP90 might be undergoing more physiological stress, resulting in decreased growth.

### In vitro benchmarking of ST-8666 and irinotecan in NSCLC cell models

**Fig 5:**
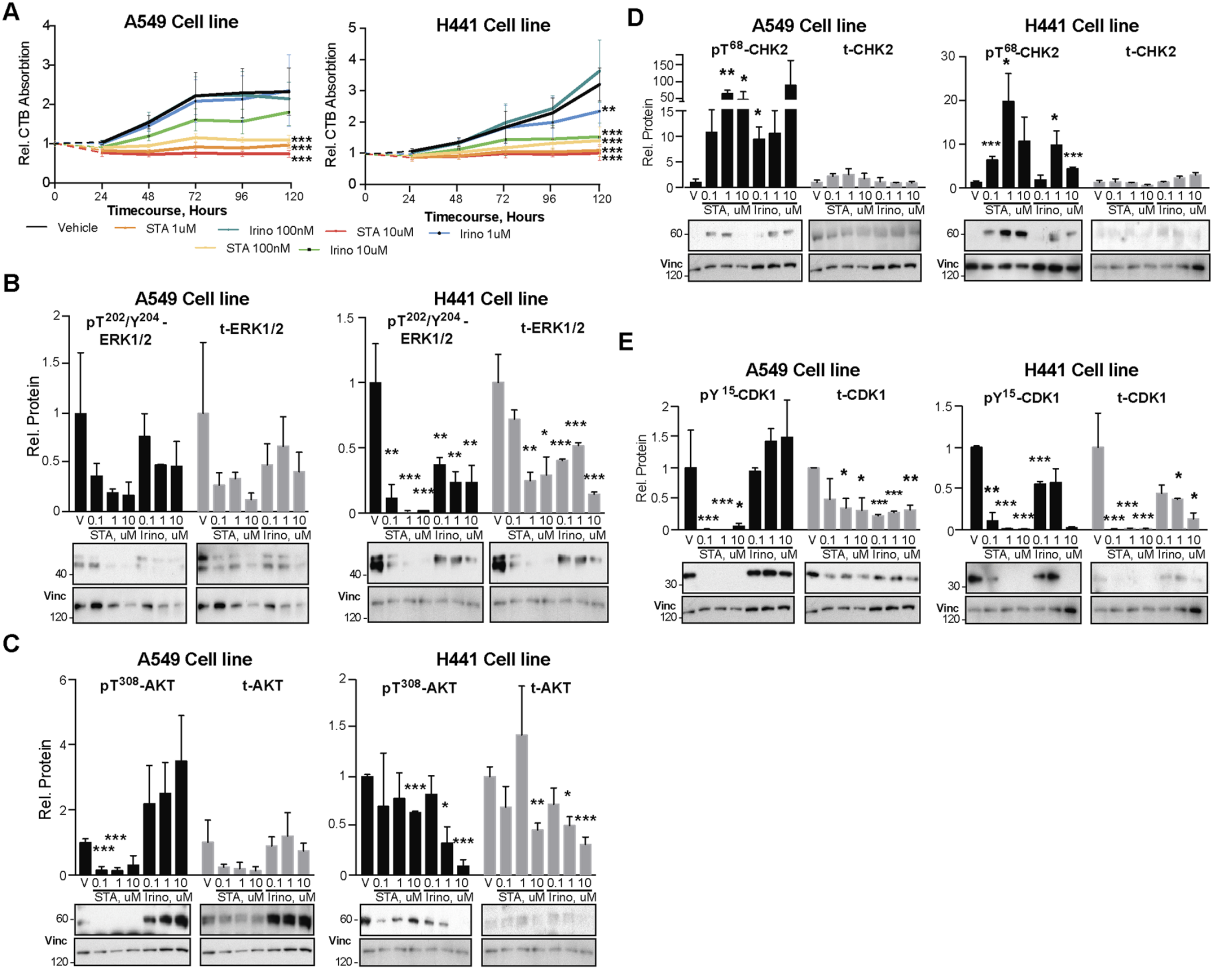
STA-8666 is more effective than irinotecan in controlling the growth of NSCLC cell lines *in vitro*. **A**. Quantification of CellTiterBlue (CTB) viability assay in human A549 and H441 NSCLC cell lines treated with vehicle, STA-8666 and irinotecan in doses of 0.1, 1, 10 μM for up to 5 days. Chart curves represent average data from two independent runs performed with duplicate samples. **B-E**. Western blot analysis of phosphorylated (p) and total (t) ERK1/2 (**B**), AKT (**C**), CHK2 (**D**) and CDK1 (**E**) protein expression in human A549 and H441 NSCLC cell lines, at 48 hours after treatment with vehicle (V), STA-8666 and irinotecan in doses of 0.1, 1, or 10 μM, as indicated. Histograms represent data from two independent experiments. All graphs: *, p≤0.05; **, p≤0.01; ***, p≤0.001 relative to vehicle controls.

To extend the *in vivo* studies, we performed a series of *in vitro* experiments using two established NSCLC cell lines, A549 and H441, to further explore consequences of treatment with STA-8666 for proliferation, cell death, and EMT, benchmarking results to treatment with irinotecan or vehicle. Treatment with STA-8666 more significantly reduced the number of viable proliferating cells compared to irinotecan used at the same dosage, based on CellTiterBlue assays. In both cell lines, treatment with irinotecan partially limited cell viability only when used at high doses (10 μM), while STA-8666 completely eliminated cell growth at doses of 100 nM, 1 μM, and 10 μM (Fig 5A). In Western blot analysis of markers associated with tumor growth and proliferation, treatment with STA-8666 compound caused a greater inhibition of ERK1/2 activity than irinotecan in both cell lines, and a greater inhibition of AKT in A549 cells (Fig 5B and 5C). SN38 is characteristically associated with DNA damage and G2/M checkpoints [27, 28]. Treatment with both STA-8666 and irinotecan increased levels of the DNA damage checkpoint-associated phosphorylation (pT^68^-CHK2), with somewhat higher levels observed with STA-8666 than with irinotecan (Fig 5D). Strikingly, STA-8666 treatment strongly downregulated pY^15^-CDK1 48 hours post treatment, indicative of arrest at G2/M. These findings are compatible with imposition of a strong G2/M checkpoint arrest by STA-8666, with this peaking at 24–48 hours, as previously described [4, 27, 28], and much more marked for STA-8666 than observed with irinotecan (Fig 5E).

**Fig 6:**
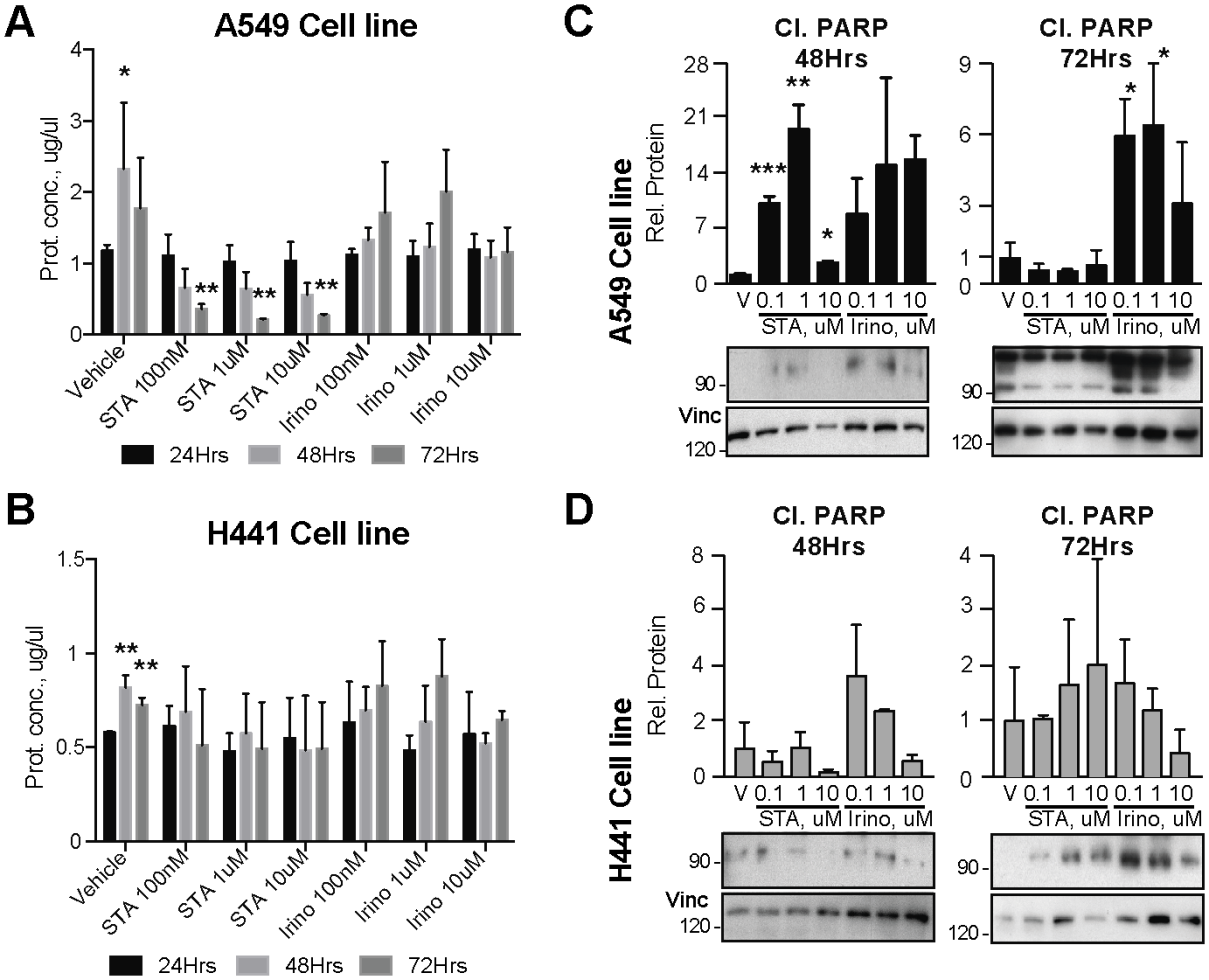
Assessment of cell detachment and cell death in STA-8666- and irinotecan-treated NSCLC cell lines *in vitro*. **A, B**. Protein concentration measurements of adherent human A549 (**A**) and H441 (**B**) NSCLC cell lines treated with vehicle, STA-8666 and irinotecan in doses of 0.1, 1, 10 μM, at 24, 48 and 72 hrs post drug treatment. Graphs represent average data from two independent runs. **C, D**. Western blot analysis of PARP cleavage in human A549 (C) and H441 (D) NSCLC cell lines, 48 and 72hrs after treatment with vehicle (V), STA-8666 or irinotecan at doses of 0.1, 1, or 10 μM, as indicated. Histograms represent data from two independent experiments. All graphs: *, p≤0.05; **, p≤0.01; ***, p≤0.001 relative to vehicle controls.

Observation of cultures treated with STA-8666 or irinotecan qualitatively revealed difference in response between A549 and H441, with STA-8666 treatment causing a high level of cell detachment in A549 cells (with > 80% cells floating). This correlated with a much lower level of protein concentration being prepared from residual adherent cells by 48 or 72 hours after treatment with this drug (Fig 6A and 6B). Both STA-8666 and irinotecan induced PARP cleavage in treated cells. For irinotecan, significant PARP cleavage was consistently observed at 72 hours in either cell line. For A549, some PARP cleavage was noted at 48 hours in H441 cells, but little at 72 hours (Fig 6C and 6D). This reduced signal at later time points likely reflected the detachment of the bulk of the cell population expressing cleaved PARP, and was compatible with faster acting dynamics of this drug, matching some prior reports [4, 5, 29].

**Fig 7:**
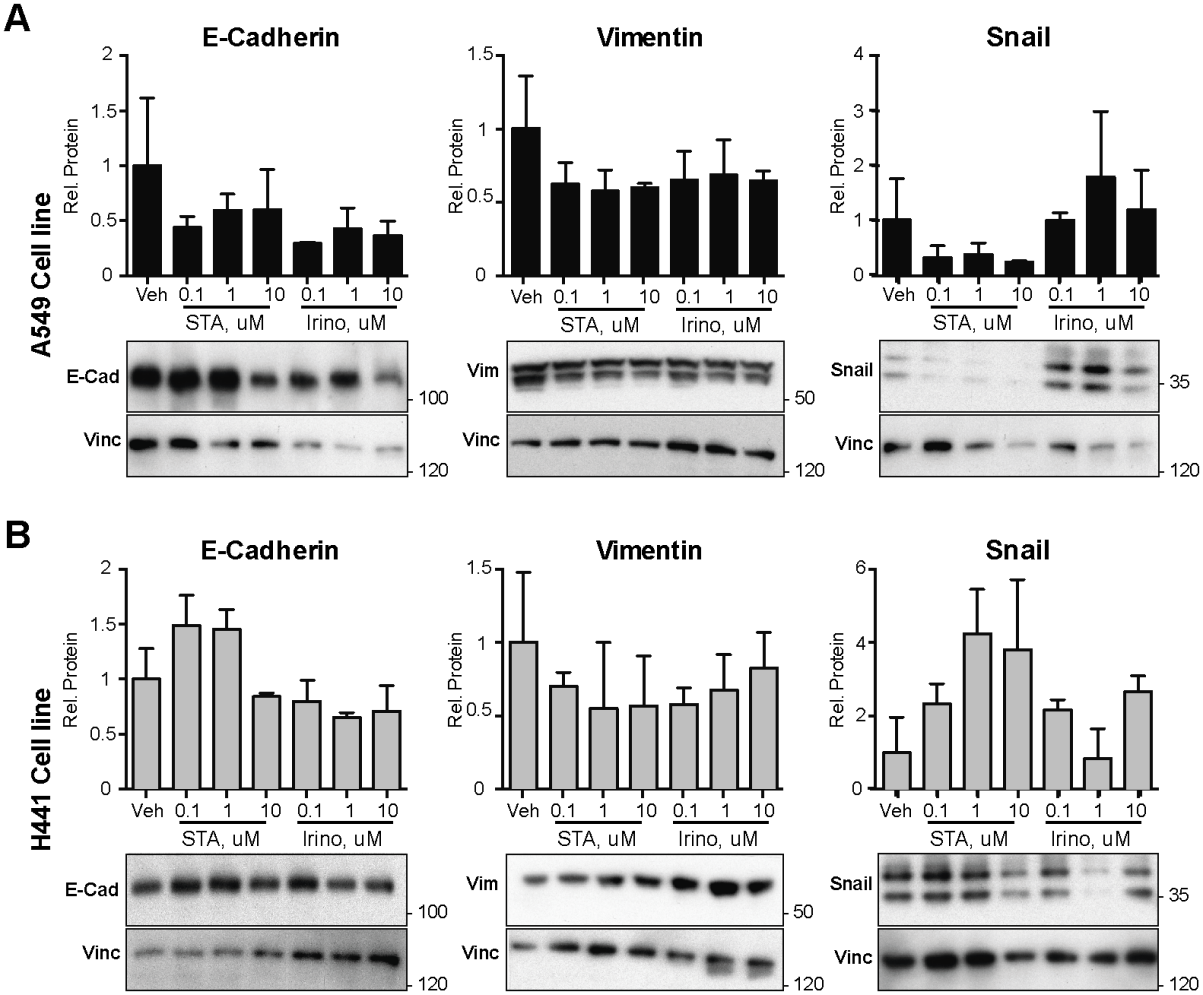
*In vitro* benchmarking of STA-8666 and irinotecan for EMT-related markers. **A, B**. Western blot analysis of expression of E-cadherin, vimentin, and Snail in human A549 (**A**) and H441 (**B**) NSCLC cell lines 48 hours after treatment with vehicle (V), STA-8666 or irinotecan at the doses of 0.1, 1, 10 μM, as indicated. Histograms represent data from two independent experiments, normalized to vehicle (V) controls.

Finally, we used Western blot analysis to evaluate whether there were differences in expression of three proteins linked to differences in epithelial or mesenchymal status (Fig 7). We assessed levels of E-cadherin, associated with epithelial identity, and vimentin and the transcription factor Snail, associated with mesenchymal identity. No significant changes were seen with either drug for vimentin or E-cadherin expression, in either cell line. STA-8666 treatment moderately affected the expression of Snail, but the direction of effect was opposite in A549 versus H441 cells, and the degree of effect did not achieve statistical significance.

## Discussion

In summary, this study for the first time demonstrates that STA-8666 is active in early stage NSCLC, very significantly reducing the growth rate and number of these tumors, via a mechanism involving induction of DNA damage and restriction of tumor proliferation. Importantly, it also suggests that STA-8666 effects are predominantly limited to tumor tissue, as no induction of DNA damage signaling or apoptosis was observed in normal lung tissue. It further demonstrates that sustained weekly dosing with STA-8666 over a period of 4 months, commencing in young mice, is not associated with any discernible sign of toxicity. These results complement recent publications on STA-8666 using short term dosing of xenograft models for other cancer types, including bladder, pancreatic, pediatric sarcoma, and triple negative breast cancer [4, 5, 29, 30] in supporting the further clinical development of this compound.

The most pronounced *in vivo* signaling effect we observed for STA-8666 was the induction of KAP1 phosphorylation. In contrast, STA-8666 modestly reduced ERK1/2 phosphorylation, and did not influence the appearance of vimentin-positive cells, associated with mesenchymal status. These data support initial preclinical mechanistic analyses of the STA-8666 compound [4, 5], which emphasized the main anti-tumor activity of this drug was the intratumoral release of a DNA damaging agent, rather than inhibition of HSP90 signaling. Our data also indicated that HSP90 expression was not depressed by treatment with STA-8666: rather, higher HSP90 levels were detected in the smaller, least proliferative tumors. In further studies performed *in vitro*, benchmarking of STA-8666 to irinotecan in NSCLC models showed similar or greater efficacy in inhibiting ERK1/2 activation, and a stronger phenotype of induction of checkpoints associated with DNA damage, and confirmed lack of an effect on epithelial or mesenchymal status.

In recently published preclinical studies, STA-8666 was much more effective than standard chemotherapeutic agents including topotecan and irinotecan (both of which also derive potency from an SN38 moiety), etoposide, and carboplatin in controlling the growth of xenografts of a number of aggressive tumor types [4, 5]. Initial studies of STA-8666 in advanced or treatment-resistant tumors of various types indicated this drug strongly induced tumor signaling associated with DNA damage response (DDR) [4, 5]. However, importantly, the initial studies typically employed STA-8666 in short term dosing regimens due to the aggressive and advanced nature of the tumors treated [4], leaving the question of toleration of long-term dosing unaddressed. Our study indicates that this drug is tolerated well over many months in young mice. This may reflect pharmacokinetic and pharmacodynamic studies performed in xenograft tumor models, which indicated a very high level of retention of STA-8666 in tumor, as opposed to normal, tissue or plasma [4, 5].

We note that while many factors go into the decision-making process for the clinical management of patients with co-morbidities or other issues that make them poor candidates for surgery, the risk of administering a drug that produces toxic side-effects that compromise quality of life is invariably a major concern [31]. Other approaches to limit toxicity of chemotherapy have included antibody-targeted cytotoxic agents; however, these are more costly to produce than small molecule agents, and can be difficult to optimize, given issues related to tumor penetrance [32, 33]. Efficiently tumor-targeted small molecule cytotoxic therapies hold the promise of ameliorating these toxicities while improving tumor control, and could result in clinically significant improvements in both progression-free and overall survival. Finally, based on its ability to induce high levels of DNA damage selectively in tumor tissue, an interesting possibility is that STA-8666 may be a candidate for use in neoadjuvant or adjuvant therapy as a radiation-sensitizer in early stage NSCLC tumors, to reduce side effects that impair quality of life [34].

## Supporting information

S1 Fig Signaling consequences of STA-8666 treatment. **A.-D**. Representative low magnification images of IHC for Ki-67 demonstrate positive staining within the tumors areas with fewer than 0.01% of cells for Ki-67 in the lung (**A**). Values of positive staining below quantitation limits in the lung are also notable for cleaved caspase 3 (**B**), pS^824^-KAP1 (**C**), and pT^202^/Y^204^-ERK1/2 (**D**). Magnification: 4x. Scale bars: 1.5mm.
(TIF)

S2 Fig Statistical analysis of expression correlations in between vehicle and STA-8666 treated mice cohorts. Shown, Spearman nonparametric correlation coefficient with two-tailed p-value between the parameters indicated. Each dot represents an individual mouse. P values shown in red are for STA-8666 treatment cohort, for blue are for vehicle treatment cohort.
(TIF)
