# Supplementary figures and images for: Tumor-targeted SN38 inhibits growth of early stage non-small cell lung cancer (NSCLC) in a KRas/p53 transgenic mouse model
Source: PLoS One. 2017 Apr 28;12(4):e0176747. doi: 10.1371/journal.pone.0176747 (PMC5409145; doi:10.1371/journal.pone.0176747)

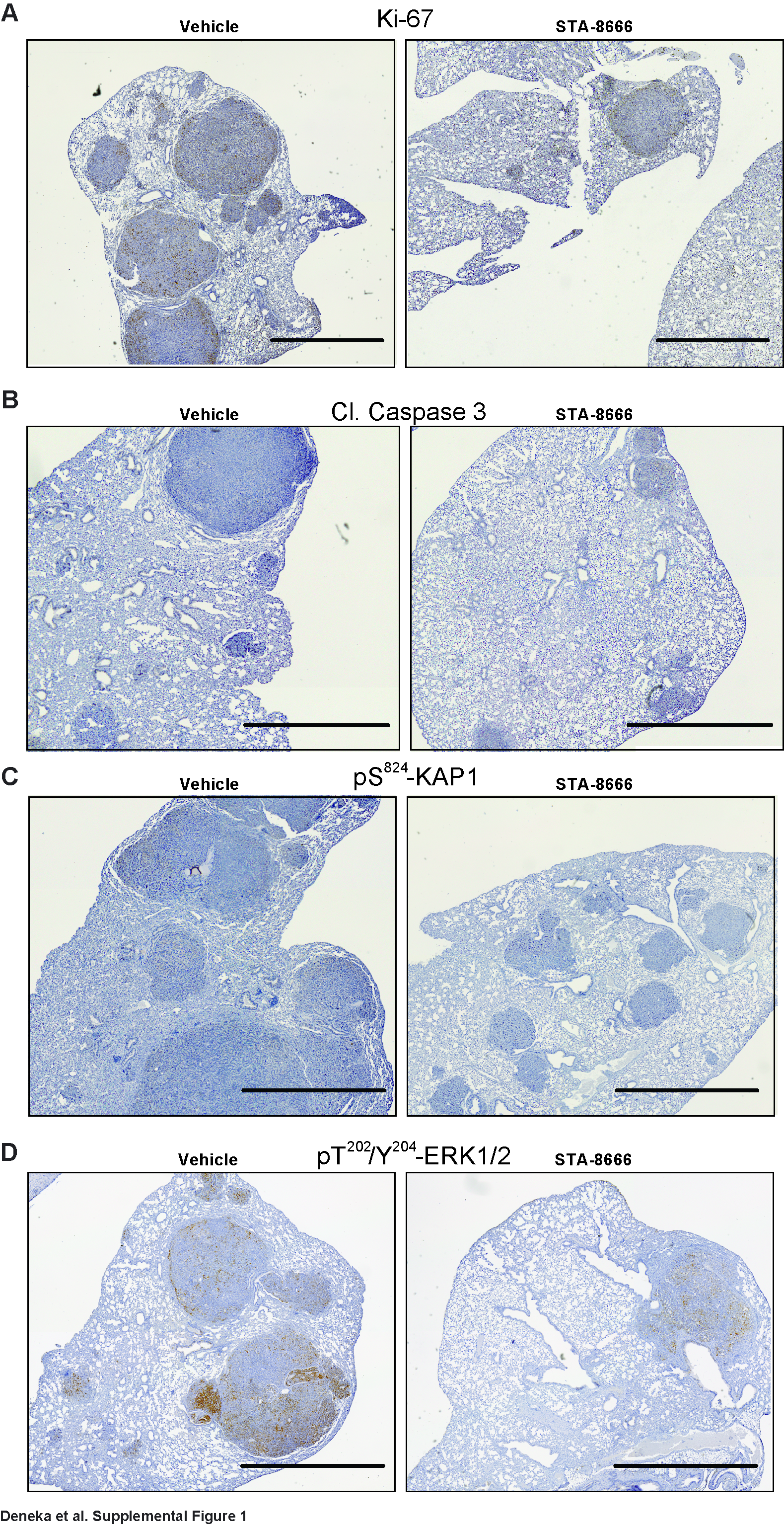

Supplement: S1 Fig — A.-D. Representative low magnification images of IHC for Ki-67 demonstrate positive staining within the tumors areas with fewer than 0.01% of cells for Ki-67 in the lung (A). Values of positive staining below quantitation limits in the lung are also notable for cleaved caspase 3 (B), pS824-KAP1 (C), and pT202/Y204-ERK1/2 (D). Magnification: 4x. Scale bars: 1.5mm. (TIF) [file pone.0176747.s001.tif]

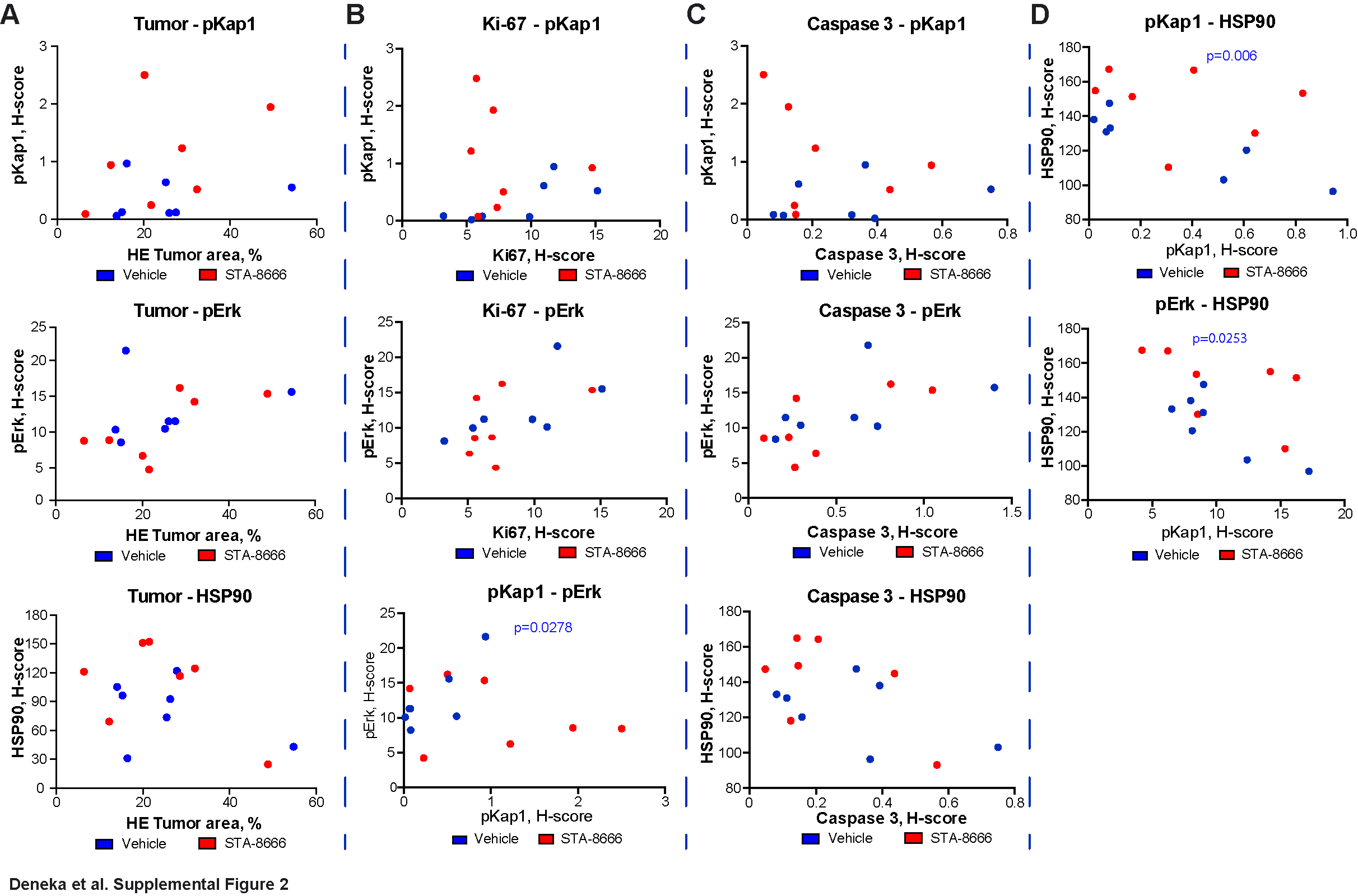

Supplement: S2 Fig — Shown, Spearman nonparametric correlation coefficient with two-tailed p-value between the parameters indicated. Each dot represents an individual mouse. P values shown in red are for STA-8666 treatment cohort, for blue are for vehicle treatment cohort. (TIF) [file pone.0176747.s002.tif]
